# Lymphocytic, cytokine and transcriptomic profiles in peripheral blood of dogs with atopic dermatitis

**DOI:** 10.1186/s12917-016-0805-6

**Published:** 2016-08-23

**Authors:** Alicja Majewska, Małgorzata Gajewska, Kourou Dembele, Henryk Maciejewski, Adam Prostek, Michał Jank

**Affiliations:** 1 Department of Physiological Sciences, Faculty of Veterinary Medicine, Warsaw University of Life Sciences-SGGW, Warsaw, Poland; 2 Department of Small Animal Diseases with Clinic, Faculty of Veterinary Medicine, Warsaw University of Life Sciences-SGGW, Warsaw, Poland; 3 Department of Computer Engineering, Wroclaw University of Technology, Wrocław, Poland; 4 Veterinary Institute, Faculty of Veterinary Medicine and Animal Sciences, Poznań University of Life Sciences, Poznań, Poland

**Keywords:** Canine atopic dermatitis, Peripheral blood, Lymphocytes, Cytokines, Microarray

## Abstract

**Background:**

Canine atopic dermatitis (cAD) is a common chronic and pruritic skin disease in dogs. The development of cAD involves complex interactions between environmental antigens, genetic predisposition and a number of disparate cell types. The aim of the present study was to perform comprehensive analyses of peripheral blood of AD dogs in relation to healthy subjects in order to determine the changes which would be characteristic for cAD.

**Results:**

The number of cells in specific subpopulations of lymphocytes was analyzed by flow cytometry, concentration of chosen pro- and anti-inflammatory cytokines (IL-4, IL-10, IL-13, TNF**-**α, TGF-β1) was determined by ELISA; and microarray analysis was performed on RNA samples isolated from peripheral blood nuclear cells of AD and healthy dogs. The number of Th cells (CD3^+^CD4^+^) in AD and healthy dogs was similar, whereas the percentage of Tc (CD3^+^CD8^+^) and Treg (CD4^+^CD25^+^ Foxp3^+^) cells increased significantly in AD dogs. Increased concentrations of IL-13 and TNF-α, and decreased levels of IL-10 and TGF-β1 was observed in AD dogs. The level of IL-4 was similar in both groups of animals. Results of the microarray experiment revealed differentially expressed genes involved in transcriptional regulation (e.g., transcription factors: *SMAD2, RORA*) or signal transduction pathways (e.g., *VEGF, SHB21, PROC*) taking part in T lymphocytes lineages differentiation and cytokines synthesis.

**Conclusions:**

Results obtained indicate that CD8^+^ T cells, beside CD4^+^ T lymphocytes, contribute to the development of the allergic response. Increased IL-13 concentration in AD dogs suggests that this cytokine may play more important role than IL-4 in mediating changes induced by allergic inflammation. Furthermore, observed increase in Treg cells in parallel with high concentrations of TNF-α and low levels of IL-10 and TGF-β1 in the peripheral blood of AD dogs point at the functional insufficiency of Treg cells in patients with AD.

**Electronic supplementary material:**

The online version of this article (doi:10.1186/s12917-016-0805-6) contains supplementary material, which is available to authorized users.

## Background

Canine atopic dermatitis (cAD) is a chronic and recurrent inflammatory and pruritic skin disease which affects 10 % of canine population. This disease is one of the most prevalent skin diseases in dogs, with characteristic clinical features most commonly associated with IgE- mediated hypersensitivity to environmental allergens. Its pathogenesis is associated with a complex of interactions between environmental factors, genetic predisposition, defective skin barrier and immunological hyperreactivity [1, 2]. AD develops as a result of defective innate and adaptive immune responses. The inflammatory reaction is caused by biphasic T cell polarization. The initial acute T-helper 2 (Th2) response is characterized by predominant secretion of interleukins: IL-4, IL-5 and IL-13 resulting in recruitment of eosinophils into inflammatory site, and activation of B lymphocytes, which are stimulated to produce IgE. The allergen-specific IgE binding to mast cells causes degranulation of these cells. The secreted inflammatory mediators lead to inflammation. In human AD (hAD) acute phase leads to Th1-driven chronic phase. The activation of Th1 cells causes IFN-γ and IL-2, IL-12 secretion [3]. In dogs only the initial Th2 type response is typically found, and it is difficult to recognize the typical Th1 type response, but rather a mixed Th1-Th2 response is observed [4, 5]. Until recently studies have been focused on the imbalance of Th1 and Th2 cells, but now in human, as well as veterinary medicine multiple lymphocyte phenotypes are considered to play a role in the immune response during AD. Th9 and Th17 cells control local tissue inflammation secreting proinflammatory cytokines and chemokines [6, 7]. In scientific literature information about the role of CD8^+^ T cytotoxic (Tc) cells is scarce. Some studies indicate that CD8^+^ T cells may contribute to development of human AD skin lesion, because they infiltrate to the skin very early, prior other leukocyte subsets [8]. However, Olivry et al. [9] and Sinke et al. [10] reported infiltration of CD4^+^ and CD8^+^ T cells in both lesional and non-lesional cAD skin, but CD4^+^ T cells was predominant in lesional skin. Regulatory T cells (Tregs) are a heterogenic subpopulation of lymphocytes. In humans two main Treg cells subsets are distinguished: naturally occurring thymic selected Tregs characterized by the expression of CD4^+^CD25^+^ Foxp3^+^ and type -1 IL-10 secreting Tregs (Trl1) induced in the periphery under tolerogenic conditions. Tregs are characterized by the ability to secrete anti-inflammatory cytokines, such as IL-10 and TGF-β, resulting in suppression of allergen induced specific T cells activation, and also suppression of effector cells of allergic inflammation, such as must cells, basophils, and eosinophils, as well as IgE production [7, 11–13]. Although many studies have been exploiting the functions of the subpopulations of Treg cells and their activity, the knowledge is still insufficient and requires further investigation. While a broad array of molecules have been implicated in Treg cell mediated suppression in vitro these pathways remain mostly unexplored in vivo*.* In the case of human Treg cells it has been shown that they are highly heterogenous and difficult to identify [14]. Moreover, rigorous analyses of the functional properties of these cells have been hindered by the lack of reliable surface markers. Natural Treg cells are arguably the best characterized Treg type in humans. In the case of cAD we know even less.

Cytokines play the major role in development, differentiation and function of cells (lymphocytes, mast cells, dendritic cells, and eosinophils), which take part in the immune response. During allergic inflammation, already secreted and newly synthesized cytokines are released contributing to the pathology observed in allergic diseases. They exert their effects by binding to specific cell surface receptors and inducing the expression of relevant target genes.

Until now research on cAD has been focused on lymphocytes and other leukocytes subsets, as well as cytokines and inflammatory mediators in regard to their contribution to the developing immune response in patients. Some studies aimed to reveal the molecular mechanism of cAD on transcriptomic level [15–17], and the majority of these studies were carried out on the lesional and non-lesional skin samples, allowing to analyze site directly affected by inflammation. On the other hand use of blood samples as the investigated material gives an overview of organism’s reaction on allergen. Furthermore, collection of blood samples is easier and less painful for a dog. Thus, in the present study we aimed to determine a comprehensive picture of the state of organism affected by cAD in order to investigate complex interactions between multiple genes and environmental factors inducing this disease. For this purpose we analyzed the profile of peripheral blood lymphocytes, plasma levels of cytokines and the transcription profile of peripheral blood nuclear cells in AD and clinically healthy dogs.

## Methods

### Animals

This study complies with national and institutional guidelines on the use of animals in clinical research according to the Polish legal act from January 21st, 2005 (Ustawa o doświadczeniach na zwierzętach z dnia 21 stycznia 2005 r. (Dz. U. z 2005 r. Nr 33, poz. 289 z późn.zm.)), concerning experiments performed on client owned animals. All dogs were patients of Small Animal Clinic at Warsaw University of Life Sciences. Before enrolling a dog into the study an informed consent from its owner was obtained and a high standard of care was adhered to throughout each examination. In the case of AD dogs research was carried out as part of routine veterinary diagnostic procedure. Dogs included in control group were blood donors from *“Milusia”* Veterinary Blood Bank who were submitted to the Small Animal Clinic for routine checkup.

Twenty privately owned dogs of various breeds, with cAD (13 females and seven males) were included in this study. The breeds were: Labrador retriever (3), Golden retriever (2), American Staffordshire terrier (2), Boxer (2), West Highland white terrier, American Bulldog, French Bulldog, Bull Terrier, Small Munsterlander, Dachshund, German shepherd, crossbreeds (3). Their age ranged between 1 and 8 years (mean age: 3.8 years). Eight healthy dogs served as a control group (3 females and 5 males), their age ranging between 3 and 8 years (mean age: 4.6 years). The following breeds were included in control group: American Staffordshire terrier (2), Labrador retriever (2), Bulldog, Great Dane, Staffordshire Bull Terrier, Weimaraner.

### cAD diagnosis and sample collection

Diagnosis of cAD was based on compatible history and clinical signs determined using Willemse and Prélaud diagnostic criteria, completed by Favrot criteria as follows: pruritus sine material, indoor lifestyle and the exclusion of other causes of pruritus ongoing for at least one year.

In all dogs with chronic pruritus other causative factors were excluded, i.e.,: skin parasites (Sarcoptic mange, Demodectic mange, flea allergic dermatitis). Bacterial pyoderma and *Malassezia dermatitis* were excluded on the basis of negative results of in vitro culture assays. The role of food antigens as a cause of the skin condition was assessed using elimination diets for 6–8 weeks. Clinical diagnosis of atopic dermatitis was confirmed by serological allergy testing (IDEXX allergic panel test) and intradermal skin testing (Artuvetrin test set, Netherlands). No anti-inflammatory drugs were given for at least 3 weeks prior serological test and intradermal test.

All dogs, which were classified to the investigated group had positive reactions in serological allergy testing and intradermal skin testing. Peripheral blood samples were collected just before the dogs were subjected to intradermal skin test, thus at the stage when clinical signs of AD were visible. Hematological, morphological and biochemical blood tests were conducted on samples of qualified patients. Each dog with AD, as well as the animals included in control group showed morphological parameters of blood within the reference value range.

The blood which was designated to the cytometric or transcriptomic analyses and ELISA tests was collected once, and separated into portions which were then used in particular analyses. The blood samples were collected from client-owned dogs during routine veterinary examinations.

### Blood sampling and separation of peripheral blood mononuclear cells (PBMC)

Peripheral blood was collected into EDTA anticoagulant tubes. PBMC were isolated from whole blood by density gradient centrifugation in histopaque-1077 using a protocol provided with ACCUSPIN System-HISTOPAQUE-1077 (Sigma-Aldrich, St. Louis, MO, USA).

### Analysis of lymphocyte subpopulations by flow cytometry

The lymphocyte subpopulations were analyzed by flow cytometry (FACS Aria II, BD Bioscience, San Jose, CA, USA). Two commercially available sets of antibodies (Dog T Lymphocyte cocktail and Dog Activated T Lymphocyte Cocktail, BD Pharmingen™, USA) were used to determine the number of T, Th, Tc, activated T cells and B lymphocytes. Dog T Lymphocyte cocktail comprised: APC-conjugated anti-CD3; PE-conjugated anti-CD4 and FITC-conjugated anti-CD8 antibodies. Dog Activated T Lymphocyte cocktail included 3 antibodies, namely: APC-conjugated anti-CD3; FITC-conjugated clone CTL 2.58 generated by using whole cell immunizations of IL-2 dependent feline T cell lines stimulated with PHA and Con A and reacting with dog T cell activation marker; and PE-conjugated LSM 11.425 antibody generated against cells derived from canine peripheral lymph nodes and used as a prognostic tool in dog B cell lymphoma studies. Freshly isolated PBMC were suspended in 100 μL of phosphate buffered saline (PBS) and 10 μL of appropriate antibodies were added. PBMC were incubated at room temperature for 30 min in dark, next the cells were washed in PBS and analyzed using BD FACSAria™ II flow cytometer (BD Biosciences, USA). Data were collected from 20,000 lymphocytes. The population of lymphocytes was first gated based on morphological characteristics: forward scatter (FSC) and side scatter (SSC) (gate P1). Cells located in gate P1 were then analyzed in regard to their positive staining with appropriate antibodies. Unstained cells were used as negative control. The results of T and B cells were expressed as the percentage of cells within the gating area of lymphocytes (P1) and the results of Th and Tc cells are presented as the percentage of CD3^+^ cells. The CD4^+^/CD8^+^ ratio was calculated based on the number of lymphocytes expressing CD3^+^CD4^+^ markers vs. the number of cells expressing CD3^+^CD8^+^ markers.

### Treg cells analysis by flow cytometry

In order to determine the number of Treg lymphocytes freshly isolated PBMC were first stained for 30 min with antibodies against two surface markers: APC-conjugated anti-CD4 monoclonal antibody (mAb, clone: YKIX302.9; eBioscience, San Diego, CA) and FITC-conjugated anti-canine CD25 mAb (clone: P4A10; eBioscience, San Diego, CA). Appropriate isotypic controls (Rat IgG2a: APC, Mouse IgG1:FITC) were used as negative controls. Then, cells were permeabilized in fixation/permeabilization buffer for 18 h at 4 °C in the dark. After incubation cells were stained intracellularly for Foxp3 for 30 min using cross-reactive, directly conjugated anti-mouse/rat Foxp3 PE mAb (clone: FJK-16 s; eBioscience, San Diego, CA) or isotype control (Rat IgG2a: PE). Fixation and permabilization of cells was performed using a set of buffers (Foxp3/Transcription FactorStaining Buffer Set, eBioscience, San Diego, CA) recommended by the producer for (eBioscience) Foxp3 staining. The stained cells were analyzed using flow cytometry. The population of lymphocytes was first gated on the basis of FSC vs SSC. The percentage of CD4^+^CD25^+^Foxp3^+^ (Treg) cells was quantified within the population of lymphocytes positively stained with anti-CD4^+^ antibody.

### Measurement of cytokine concentration in plasma by ELISA

The concentration of cytokines: IL-2, IL-4, IL-10, IL-13, TNF-α TGF-β 1and IFN-γ in plasma were determinated by ELISA. For all cytokines except IFN-γ dog specific tests from USCN Life Science (CLOUD-CLONE CORP., Wuhan, China) were used, and IFN-γ concentration was determined using Canine IFN-gamma Quantikine ELISA Kit (R&D System, Minnesota, USA). The detection limit was 5.6 pg/mL for IL-13, 5.6 pg/mL for IL-4, 5.8 pg/ml for IL-2, 5.9 pg/ml for IL-10, 6.2 pg/ml for TNF-α and TGF-β 1, 60 pg/ml for IFN-γ. ELISAs were performed according to the instructions of the kits producers.

Statistical analyses of the results of flow cytometric and ELISA analyses were performed using GraphPad Prism version 5.00 (GraphPad Software, Inc., USA). Statistical significance was calculated by unpaired *t*-test. Results with *P* value ≤0.05 were considered significant.

### Microarray analysis

Blood samples were collected from AD and healthy dogs into Rneasy Protect Animal Blood Tubes (Qiagen, USA). Total RNA from peripheral blood nuclear cells was isolated using a Rneasy Protect Animal Blood Kit (Qiagen, USA). Additionally, contamination with DNA was eliminated by DNAse I digestion included as an additional step of the isolation protocol. The quantity of RNA was measured by NanoDrop (NanoDrop Technologies, USA). The analysis of final RNA quality and integrity was performed using Agilent 2100 Bioanalyzer (USA) and RNA 6000 Nano Kit (Agilent, Germany). To ensure optimal data quality, only RNA samples with RIN number ≥7.8 were included in the analysis.

The analysis of gene expression profile was performed using Canine (V2) Gene Expression Microarray, 4x44K (Agilent Technologies, USA) and Agilent Technologies Reagent Set according to the manufacturer’s procedure. RNA Spike In Kit (Agilent Technologies, USA) was used as an internal control, the Low Input Quick Amp Labeling Kit was applied to amplify and label (Cy3 or Cy5) target RNA to generate complementary RNA (cRNA) for oligo-microarrays. Gene Expression Hybridization Kit was used to fragmentation and hybridization and Gene Expression Wash Buffer Kit was used for washing slides after hybridization. Acquisition and analysis of hybridization intensities were performed using Agilent DNA Microarray Scanner G2505C.

The experiment was performed using a common reference design, in which the common reference comprised a pool of equal amounts of RNA from 13 healthy dogs. These dogs did not take part in the experiment. The cRNA of common reference samples were Cy3-labelled and the cRNA of healthy dogs (control group of dogs taking part in experiment) and of AD dogs were labelled with Cy5. Twenty eight two-color microarrays were performed, one for each patient (20 microarrays with samples from AD dogs and eight from healthy dogs). On each microarray 825 ng of each sample of cRNA (Cy3-labelled common reference and Cy5-labelled control or patient) were hybridized.

### Signal detection and statistical analysis

After microarray scanning, data were extracted and background subtracted using the standard procedures contained in the Agilent Feature Extraction (FE) Software version 10.7.3.1. FE performs Lowess normalization.

Prior the analysis of differential gene expression, non-specific filtering was performed. Transcripts without expression were removed. In addition, transcripts whose median of signal in channel R and G (calculated in investigated and control samples) did not exceed 100 were identified and eliminated. This reduced the number of transcript down to 20,188.

The log2-ratio of the sample to common reference signal was calculated and the data were median-centered among all microarrays. The analysis of differential expression was performed using Limma’s method (linear methods for microarrays) and R/Bioconductor package. A multiple testing correction was applied using Benjamini and Hochberg False Discovery Rate (FDR). Microarray data were deposited at the Gene Expression Omnibus data repository under the number GSE76119.

To identify signaling pathways and gene function the microarray data was analyzed using Pathway Studio 11 (Ariadne Genomics). This is a database consisting of millions of individually modeled relationships between proteins, genes, complexes, cells, tissues and diseases [18].

### Real-time RT-PCR

To verify microarray results, the expression of three genes: *PIAS1* (protein inhibitor of activated STAT,1), *RORA* (RAR-related orphan receptor A) and *SH2B1* (SH2B adaptor protein 1) was analyzed using real-time PCR. The sequences of chosen genes were obtained from Ensembl or NCBI database. Primers were designed using Primer-Blast software (NCBI database) and verified using Oligo Calc: Oligonucleotide Properties Calculator (free software available online, provided by Northwestern University) to exclude sequences showing self-complementarity. To reduce chances of amplifying traces of genomic DNA, the primers were positioned in different exons. The secondary structures of the amplicon were examined using m-fold Web Server (free on-line access). Reference genes: *GAPDH* and *RPS19* were amplified using primer sequences previously published [5, 19, 20]. All primer sequences are listed in Table 1. Total RNA was reverse transcribed to first strand complementary DNA (cDNA) using the High Capacity cDNA Reverse Transcription (Applied Biosystems, USA). All analyses were performed on individual samples of total RNA using SYBR Select Master Mix (Applied Biosystems, USA) on Stratagene Mx3005P Quantitative PCR instrument for RT-PCR, following the manufacturer’s protocol. For all genes annealing temperature was 58 °C. The relative expression of the target gene was quantified as mean of triplicate measurements for each biological sample. Results were calculated using the 2^-ΔΔCT^ method [21].

**Table 1:** Primer sequences for real-time PCR verification of microarray result

Gene	Forward primer (5'–3')	Revers primer (5'–3')	NCBI accession number
*PIAS1*	TGGAGTTGATGGATGCTTGAG	GGACACTGGAGATGCTTGAT	transcript variant X1-X2: XM_005638536.2 5 XM_535524.5
*RORA*	AAGGCTGCAAGGGCTTTTTC	CTGCGTACAAGCTGTCTCTT	transcript varian X1- X3: XM_014109378.1 XM_535503.6 XM_014117330.1
*SH2B1*	CGTCCTCACTTTCAACTTCCA	GACACGACATAGCTGACAAGA	transcript variant X1-X8 XM_005621372.2 XM_005621371.2 XM_005621373.2 XM 005621374 XM_014114512.1 XM_014114513.1 XM_014114514.1 XM_014114515.1
*GAPDH*	GGAGAAAGCTGCCAAATATG	ACCAGGAAATGAGCTTGACA	NM_001003142.1
*RPS19*	CCTTCCTCAAAAAGTCTGGG	GTTCTCATCGTAGGGAGCAAG	XM_533657.3

## Results

### Lymphocyte subpopulations in peripheral blood of AD and healthy dogs

The number of cells in specific subpopulations of lymphocytes in peripheral blood of dogs with atopic dermatitis and control group (healthy dogs) was analyzed using flow cytometry, and are presented in Table 2. The mean percentage of lymphocytes was similar in both groups: 45.2 ± 3.3 (AD dogs) and 42.7 ± 6.5 (control). The percentage contribution of T cells (CD3^+^) and B cells in lymphocytes population was significantly smaller in AD dogs than in control dogs (*P* = 0.04). The number of CD3^+^CD4^+^ cells within the population of T lymphocytes was almost the same in both investigated groups; whereas the percentage of CD3^+^CD8^+^ T cells was significantly higher in AD dogs than in control group (*P* = 0.002). The CD4^+^/CD8^+^ cells ratio was significantly lower (*P* = 0.02) in AD dogs than in control animals. The number of CD4^+^CD25^+^ Foxp3^+^ Treg cells was increased in AD dogs in comparison to the healthy ones (*P* < 0.0001).

**Table 2:** Percentage of lymphocyte subsets in peripheral blood of dogs with atopic dermatitis and healthy dogs

Lymphocytes subpopulations	% of cells in different subpopulations of white blood cells		
	AD dogs	Control dogs	*P* value
Lymphocytes	45.2 ± 3.3	42.7 ± 6.5	0.7147
CD3^+^	25.67^*^ ± 3.02	35.90 ± 2.65	0.0408
B cells	3.81^*^ ± 0.68	6.24 ± 0.82	0.0373
CD3^+^ CD4^+^	67.40 ± 2.01	70.70 ± 1.94	0,1866
CD3^+^CD8^+^	19.56 ^**^ ± 0.87	14.71 ± 1.11	0.0023
CD4^+^/CD8^+^	3.86^*^ ± 0.33	5.31 ± 0.41	0.0132
CD4^+^CD25^+^ Foxp3^+^	1.54^***^ ± 0.18	0.48 ± 0.08	<0.0001

The results are expressed as mean percentage of positive cells ± SEM, ratio CD4^+^/CD8^+^ was calculated as the ratio of absolute number of cells within the T lymphocytes population, symbol ^*^represents the level of significance: *P* ≤ 0.05, **P ≤ 0.01, ***P ≤ 0.001

### Cytokine profile in peripheral blood of AD and healthy dogs

**Fig. 1:**
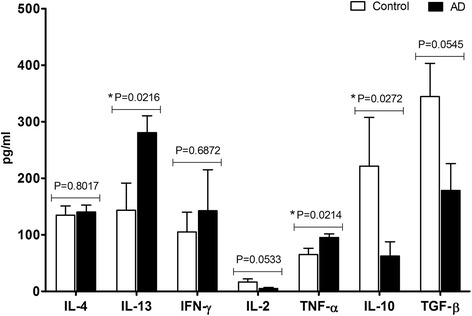
Concentration of cytokines in plasma of dogs with atopic dermatitis and healthy dogs. Symbol * represents the level of significance: *P* ≤ 0.05

The level of cytokines in plasma of AD and healthy dogs was determined based on ELISA tests, and the results obtained are presented in Fig. 1. In four out of seven analyzed cytokines significant differences were observed between the two groups of dogs. The level of IL-13 and TNF-α was significantly higher in AD dogs than in controls (*P* = 0.02); whereas L-10 was significantly lower in AD patients (*P* = 0.03). No differences were noted in the case of IL-4. In most patients IFN-γ was undetectable, only in two dogs from each investigated group this cytokine was detected on quite high level. Likewise, cytokine IL-2 was not detected in all patients, and the concentration of this interleukin was lower in AD than in control dogs (*P* = 0.05). It should be noted that IL-2 was detected in 9 out of 20 AD dogs, but the values detected exceeded the detection limit of used ELISA kit (5.8 pg/ml) only in 3 samples. Although no significant difference was noted in the level of TGF-β1 between both groups of animals, there was a tendency towards lower values of concentration of this cytokine in AD dogs (*P* = 0.55). Detailed information about the range of detected levels of cytokines and the number of dogs in which cytokines were detected are presented in Additional file 1: Table S1.

### Microarrays analysis

In order to determine possible differences in gene expression in peripheral blood nuclear cells of AD and healthy dogs a microarray analysis was performed. The analysis revealed 139 differentially expressed transcripts between two investigated groups: AD and healthy dogs (FDR-adjuted *P* value =0.085). Among these 139 differentially expressed transcript only 59 genes have known ontology (Table 3). Even though none of the listed genes encoded proteins typically involved in the immune response, the transcripts showing differential expression between AD and healthy dogs were directly or indirectly connected with transcriptional regulation (*SMAD2, RORA*) or signal transduction pathways (e.g., *VEGF, SHB21, PROC*) taking part in T lymphocytes lineages differentiation and cytokines synthesis.

**Table 3:** The list of differentially regulated genes with known ontology in AD vs healthy dogs

GeneSymbol	Description	logFC	Regulation	*p*-value adj.
*NPTXR*	neuronal pentraxin receptor	−1,09	down	0,085
*CCDC54*	coiled-coil domain containing 54	−1,09	down	0,085
*UFL1*	UFM1-specific ligase 1	−0,76	down	0,085
*BBS2*	Bardet-Biedl syndrome 2	−0,68	down	0,085
*DCUN1D1*	defective in cullin neddylation 1, domain containing 1	−0,66	down	0,085
*RORA*	RAR-related orphan receptor A	−0,66	down	0,085
*PKIB*	protein kinase (cAMP-dependent, catalytic) inhibitor beta	−0,61	down	0,085
*OPN1SW*	opsin 1 (cone pigments), short-wave-sensitive	−0,58	down	0,085
*FAM49A*	family with sequence similarity 49, member A	−0,58	down	0,085
*PURA*	purine-rich element binding protein A	−0,57	down	0,085
*CA14*	carbonic anhydrase 14-like (LOC100855809),	−0,54	down	0,085
*SCN1B*	sodium channel, voltage-gated, type I, beta subunit	−0,53	down	0,085
*SCOC*	short coiled-coil protein	−0,53	down	0,085
*PIWIL4*	PIWIL4 piwi-like RNA-mediated gene silencing 4	−0,53	down	0,085
*WNT5B*	wingless-type MMTV integration site family, member 5B	−0,53	down	0,085
*FAM155B*	family with sequence similarity 155, member B	−0,52	down	0,085
*EPHB3*	EPH receptor B3	−0,52	down	0,085
*LOC100856122*	olfactory receptor 4 K5-like	−0,52	down	0,085
*BPIFB1*	BPI fold containing family B, member 1	−0,52	down	0,085
*TNKS2*	tankyrase, TRF1-interacting ankyrin-related ADP-ribose polymerase 2	−0,51	down	0,085
*RTN4RL1*	reticulon 4 receptor-like 1	−0,51	down	0,085
*TTYH2*	tweety family member 2	−0,50	down	0,085
*AK9*	adenylate kinase 9	−0,50	down	0,085
*GPR12*	G protein-coupled receptor 124	−0,49	down	0,085
*PCIA1*	cross-immune reaction antigen	−0,49	down	0,085
*VEGFA*	vascular endothelial growth factor A	−0,49	down	0,085
*OTOP1*	otopetrin 1	−0,49	down	0,085
*TTF2*	transcription termination factor, RNA polymerase II	−0,49	down	0,085
*FGFBP3*	fibroblast growth factor binding protein 3	−0,48	down	0,085
*ABHD15*	abhydrolase domain containing 15	−0,48	down	0,085
*PROC*	protein C (inactivator of coagulation factors Va and VIIIa)	−0,47	down	0,085
*EM2*	transmembrane protein 25	−0,47	down	0,085
*EMC1*	ER membrane protein complex subunit 1	−0,47	down	0,085
*TMEM52*	transmembrane protein 52	−0,47	down	0,085
*RHOV*	ras homolog family member V	−0,47	down	0,085
*TEKT1*	tektin 1	−0,47	down	0,085
*GDPD3*	glycerophosphodiester phosphodiesterase domain containing 3	−0,47	down	0,085
*ANAPC5*	anaphase promoting complex subunit 5 [	−0,47	down	0,085
*ABCA4*	ATP-binding cassette, sub-family A (ABC1), member 4	−0,46	down	0,085
*ZC3H10*	zinc finger CCCH-type containing 10	−0,46	down	0,085
*SMAD2*	SMAD family member 2	−0,46	down	0,085
*SSH3*	slingshot protein phosphatase 3	−0,46	down	0,085
*SH2B1*	SH2B adaptor protein 1	−0,45	down	0,085
*PIAS1*	protein inhibitor of activated STAT, 1	−0,45	down	0,085
*SUFU*	suppressor of fused homolog (Drosophila)	−0,44	down	0,085
*ALKBH4*	alkB, alkylation repair homolog 4 (E. coli) A	−0,44	down	0,085
*NRG2*	neuregulin 2	−0,44	down	0,085
*TMEM231*	transmembrane protein 231	−0,44	down	0,085
*OGT*	OGT O-linked N-acetylglucosamine (GlcNAc) transferase	−0,44	down	0,085
*RBM25*	RNA binding motif protein 25	−0,43	down	0,085
*SCN2B*	sodium channel, voltage-gated, type II, beta subunit	−0,42	down	0,085
*PTP4A2*	protein tyrosine phosphatase type IVA, member 2	−0,42	down	0,085
*PEX26*	peroxisomal biogenesis factor 2	−0,41	down	0,085
*EIF4ENIF1*	eukaryotic translation initiation factor 4E nuclear import factor 1	−0,40	down	0,085
*THEMIS*	thymocyte selection associated	−0,39	down	0,085
*ERLIN1*	ER lipid raft associated 1	−0,39	down	0,085
*DDX1*	DEAD (Asp-Glu-Ala-Asp) box helicase 1	−0,39	down	0,085
*PABPC5*	poly(A) binding protein, cytoplasmic 5	−0,37	down	0,085
CDT1	chromatin licensing and DNA replication factor 1	−0,34	down	0,085

Log fold change (FC) expressed as log2

*p*-value adj. was adjusted by multiple testing using the Benjamini-Hochberg False discovery rate procedure (FDR) for each comparison

### Validation of microarray data

**Fig. 2:**
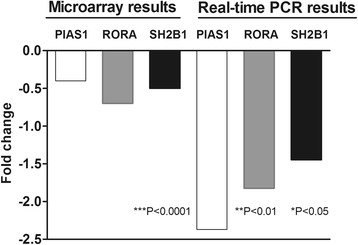
Expression of *PIAS1, RORA, SH2B1* genes in peripheral blood nuclear cells of AD and healthy dogs analyzed using microarray and real-time PCR

To validate the microarray data we selected genes which were shown to be directly or indirectly involved in regulation of T lymphocytes differentiation, and synthesis of investigated cytokines. Thus, we chose to analyze the expression of *PIAS1, RORA* and *SH2B1* genes using real-time qPCR. The results obtained confirmed the decreased expression of all tested genes in AD dogs in comparison to healthy animals (Fig. 2).

## Discussion

In the present study we aimed to perform comprehensive analyses of peripheral blood of AD dogs in relation to healthy subjects in order to determine the changes which would be characteristic for cAD. Therefore we analyzed the number of cells in specific subpopulations of lymphocytes, determined the concentration of chosen pro- and anti-inflammatory cytokines, and performed a microarray analysis to determine the gene expression profile of peripheral blood nuclear cells in both groups of dogs. Flow cytometric analyses revealed that the percentage contribution of CD3^+^ T cells and B lymphocytes in AD dogs was significantly lower than in healthy ones, the number of CD3^+^CD4^+^ T cells in both groups was similar, and the percentage of CD3^+^CD8^+^ lymphocytes increased significantly in AD dogs. These results did not confirm previously published reports demonstrating that patients with cAD have increased number of both CD3^+^CD4^+^ and CD3^+^CD8^+^ T cells, since in our study only the subpopulation of Tc lymphocytes was increased. However, the majority of studies on dogs have focused on lymphocytes which infiltrate the atopic skin, showing the pivotal role of Th1 and Th2 cells in the development of skin inflammation. It was demonstrated that in lesional and non-lesional atopic skin the percentage of CD3^+^, CD4^+^ and CD8^+^ cells increased in comparison with the skin of healthy dogs, but the presence of B cells was scarce and detected only in lesional skin [9]. In canine lesional atopic skin, the predominat type of T lymphocytes was CD4^+^ cells; whereas, in non-lesional atopic skin an infiltration of both CD4^+^ and CD8^+^ T-cells was observed, without predominance of CD4^+^ T cells [9, 10]. Hennino et al. [8, 22] reported that CD8^+^ cells were essential for the development of AD skin inflammation in both mice and humans. In these studies AD was provoked by the epicutaneos application of house dust mites (HDM). The authors observed recruitment of CD8^+^ lymphocytes to the *Drematophagoides farine* exposed skin before the infiltration of other leukocytes subsets. Also in lesional skin of dogs with cutaneous adverse food reaction (CAFRs) increased number of CD3^+^CD4^+^ and CD3^+^CD8^+^ cells was observed in comparison to skin of control dogs. Furthermore, CD3^+^CD8^+^ phenotype predominated over CD3^+^CD4^+^ phenotype in these patients. The study on murine model indicated dual time-dependent role of CD8^+^ T cells in development of airway hyperreactivity (AHR) [23]. The authors observed that at early stage the CD8^+^ T cells protected the organism from systemic sensitization, but when systemic sensitization was established, these cells may play a bystander or proinflamatory role in the development of allergic airway disease [23]. There is only a few reports regarding the subsets of lymphocytes in peripheral blood in atopic dogs. Tarpatakiet al. [24] indicated an increase of CD4^+^/CD8^+^ ratio of T lymphocytes in AD dogs in comparison with healthy dogs. In contrast, other studies obtained results which are comparable to our data showing that the percentage of CD4^+^ T cells did not change in atopic dogs in relation to healthy dogs but percentage of CD8^+^ T cells significantly increased and as a result ratio of CD4^+^/CD8^+^ T lymphocytes in AD dogs decreased [25]. In German shepherd dogs suffering from pyoderma (GSP) the percentage of CD4^+^ cells decreased, CD8^+^ cells increased and ratio CD4^+^/CD8^+^ decreased in comparison to healthy dogs [26]. Thus, the results showing a marked increase in CD8^+^ T cells in peripheral blood of AD dogs suggest that these lymphocytes may play an important role in skin inflammation during AD.

We also observed a significant increase in the percentage of Treg cells in dogs with AD in comparison with healthy controls. Subsets of Treg cells are responsible for healthy immune response to allergens. These cells mediate the peripheral tolerance to allergen suppressing proliferation and activity of effector cells. The population of Treg cells is heterogeneous by the expression of various surface markers and can be subdivided into several subtypes. The most known subset called naturally occurring regulatory T cells (nTreg cells) was defined based on expression of surface CD25 (IL-2Rα) and transcription factor Foxp3. It was demonstrated that in dogs CD4^+^CD25^+^ Foxp3^+^ cells were able to suppress the proliferation of responder CD4^+^ T cells in vitro [27]. Our study demonstrated that the percentage of circulating CD4^+^CD25^+^ Foxp3^+^ cells in atopic dogs was significantly higher compared to healthy dogs, and these results were similar to recent data of other research groups [28, 29]. On the contrary other investigations have indicated that the Treg cells are not fully efficient in atopic patients in comparison with healthy individuals. In vitro studies on T lymphocytes isolated from human grass allergic donors showed that Treg cells from these donors failed to inhibit proliferation but not cytokine production of CD4^+^CD25^−^ T cells at high antigen doses, while Treg from non-atopic donors retained their regulatory properties [30]. It is possible, however, that the higher percentage of Tregs detected in AD patients in our study could be related to the chronic nature of the disease [28].

It has been shown that atopic dermatitis is affected by aberrant immune response and that imbalance in the T lymphocytes population and associated cytokine pattern play a crucial role. Cytokines are very powerful messengers, which control regulatory system in all levels: production, secretion, effect on target. During allergic inflammation, preformed and newly synthesized cytokines are released and contribute to the pathological response to allergen. These cytokines have a wide range of activities on different cell types. In our study we determined the plasma level of a few cytokines which take part in development of atopy and maintain the inflammatory state. In the initial acute phase of AD eosinophils and Th2 cells are the predominant subpopulation of immune cells, and increased production of IL-4, IL-5, IL-13 is observed. In chronic AD lesions there is a switch towards Th1 cells secreting IFN-γ and IL-12 and also IL-2. In some studies it was indicated that chronic phase is not characterized only by the presence of Th1 cells but rather by a mixed Th1 and Th2 profile [4, 5]. It results from a dynamic nature of this process.

In our study it is difficult to define the phase of immune response. The pattern of cytokines’ concentration in plasma partially indicates the domination of Th-2 cells subpopulation, but the interpretation is not obvious. We noted a significantly higher level of IL-13, and slightly elevated concentration of IL-4 in AD dogs in comparison to healthy controls. Similar results were presented by Schlotter [5], who observed increased expression of IL-13 mRNA in lesional skin and non lesional skin of AD dogs, but an unchanged expression of *IL-4* gene. Increase expression of IL-13 mRNA was also noted in the skin of dogs challenged with house dust mites (HDM) allergen for 24 and 48 h, while the expression of other interleukins secreted by Th-2 cells was low and the level did not change significantly in time [31]. In addition, in humans after HDM allergen challenge secretion of IL-13 to the peripheral blood also occurred earlier and for much longer time than in the case of IL-4 [32, 33]. These results suggest that IL-13 may play more important role in atopic response than IL-4.

In the present study IFN-γ, considered as the “canonical” Th1 cytokine, was detected only in two healthy and two AD dogs. We presume that the level of IFN-γ in plasma may be a characteristic feature of each individual. In fact, among dogs exposed to immunotherapy IFN-γ was detected only in the same two dogs in which this cytokine was detected prior therapy (data not show). Similar observations were made by Hayashiya et al. [34], who detected IFN-γ mRNA expression in PBMC of nine out of ten control dogs and in two out of eight AD dogs, and the average expression of IFN-γ mRNA was lower in AD dogs. The production of IFN-γ may be related to the stage of development of skin lesions and the type of investigated tissue. In the skin of dogs exposed to epicutaneous allergen challenge IFN-γ mRNA expression was the highest at the early time points post challenge (6 h) and after 4 days, whereas at the intermediate period the expression of this cytokine was at a low level [31]. This observation shows the dynamic nature of IFN-γ induction.

On the other hand, we noted a significantly higher concentration of TNF-α in plasma of atopic dogs then in healthy animals. This result seems to be directly connected with the state of atopic inflammation in investigated AD dogs. TNF-α is a pleiotropic cytokine, which plays a key role in bridging innate and adaptive immunity in chronic inflammatory disease [35]. Excessive secretion of this cytokine is associates with susceptibility to allergies. There are many potential sources of TNF-α: macrophages, T lymphocytes, mast cells, eosinophils and neutrophils [36]. In atopy disease TNF-α is frequently recognized as a cytokine that belongs to the Th1-type profile. TNF-α is non-specific proinflamatory mediator, inducing expression of cell adhesion molecules and eotaxins effecting recruitment of eosinophils, neutrophils, and macrophages to the sites of allergic inflammation. Furthermore, it increases the proliferation of B and T lymphocytes, and is able to induce apoptosis of keratinocytes. Additionally, TNF-α impairs the regulatory activity of natural Treg cells via the TNF-α receptor 2 (TNFR2) signaling pathway to down-modulate Foxp3 expression in allergic asthma [37]. Our results are in agreement with a previous study by Nuttall et al. [4] who observed increased expression of TNF-α mRNA in lesional skin of atopic dogs in comparison to the non-lesional and healthy control tissue.

In contrast to TNF-α concentration the level of IL-2 in plasma of AD dogs was reduced in comparison to healthy group, but the level of this cytokine was generally undetectable in majority of samples. IL-2 is secreted by Th1 cells, and it is a growth factor essential for proliferation, survival and function of both effector T cells (Teffs) and Treg cells [38–40]. Treg cells do not produce IL-2 but this interleukin is required for their activation by regulating Foxp3 expression via signaling transducer and activator of transcription 5 (STAT5) [41–44]. However, Foxp3 represses the expression of *IL-2* and activates expression of *CD25* genes (IL-2 receptor) by binding to the promoter of these genes [42–45]. Tregs also inhibit production of IL-2 in Teffs, and additionally they have a high expression of IL-2 receptors (CD25) which gives them the capacity to compete with Teffs for IL-2 [40, 44, 46, 47]. Research describing the function of IL-2 in the skin of AD dogs demonstrated that the expression of IL-2 mRNA was increased in lesional skin in comparison to non-lesional and and healthy cutaneous tissue [4]. In our study a decrease of IL-2 concentration in blood plasma of AD dogs coincided with increase number of Treg cells in these animals, suggesting the suppressive effect of Treg lymphocytes. However, AD dogs also had lower plasma levels of two anti-inflammatory cytokines: IL-10 and TGF-β1, which is contradictory to the concept of suppressive effect of the immune response. Both of these anti-inflammatory cytokines secreted by Treg cells suppress allergen-induced specific T-cell activation and allergic processes. IL-10 is synthesized by a wide range of cells besides Tregs: Th2 cells, B cells, monocytes, dendritic cells, and mast cells. It inhibits the production of proinflammatory cytokines and cytokine receptors [13, 48]. IL-10 is also a potent suppressor of allergen-specific IgE, simultaneously inducing IgG4 production by a direct influence of Tregs on B-cells. Most studies regarding cAD do not report any differences in plasma concentration of IL-10 between AD and healthy dogs [49], or in the expression of IL-10 mRNA in lesional and non-lesional skin [4], or in circulating PBMCs [34]. However, Maeda et al. [50] observed decreased *IL-10* expression in blood of cAD patients during allergen challenge, which is consistent with our results. TGF-β1 is secreted by Tregs and also similarly to IL-2 it converts naïve T cells to Treg cells by inducing the expression of Foxp3. TGF-β1 together with IL-6 contributes to the generation of Th17 cells [51]. TGF-β1 inhibits the proliferation, differentiation, and survival of both B and T lymphocytes [48]. Membrane-bound TGF-β1 shows unique and potent immunosuppressive activity towards Tregs, associated with direct contact of TGF-β1 with its receptors localized on Treg cell membrane. Upon activation of TGF-β1 receptor type II (TbRII), Treg cells become susceptible to TGF-β1 and demonstrate activation of TGF-β1 - dependent transcription factors (Smads). The Treg membrane-associated TGF-β1 might provide a sustained contact-dependent signal crucial to suppressing effector T cells and mediating cell cycle arrest, and blocking cytokine production. In addition to contact-dependent suppression of TbRII-expressing responder T cells, soluble TGF-β1 shows many direct effects on cells of the immune system, including regulation of macrophage activation, dendritic cell maturation, T-cell proliferation and cytokine generation, and B-cell antibody production [52]. Nevertheless, the role of TGF-β1 in cAD has not been fully elucidated, and the results published so far are often contradictory. Schlotter et al. [5] did not observe any difference in the expression of this cytokine in lesional and non-lesional skin of AD dogs as well as control skin samples. On the other hand Nuttall el al. [4] demonstrated lower expression of TGF-β1 in lesional and non-lesional skin in comparison to control tissue. In blood of patients undergoing allergen challenge the level of TGF-β1 mRNA decreased after four days, which is similar to our observations [50]. Findings of the present study indicate that despite increased number of Treg cells (CD4^+^CD25^+^ Foxp3^+^) detected in AD dogs the level of anti-inflammatory cytokines produced by these cells was insufficient to protect the organism against the pathological immune response.

**Fig. 3:**
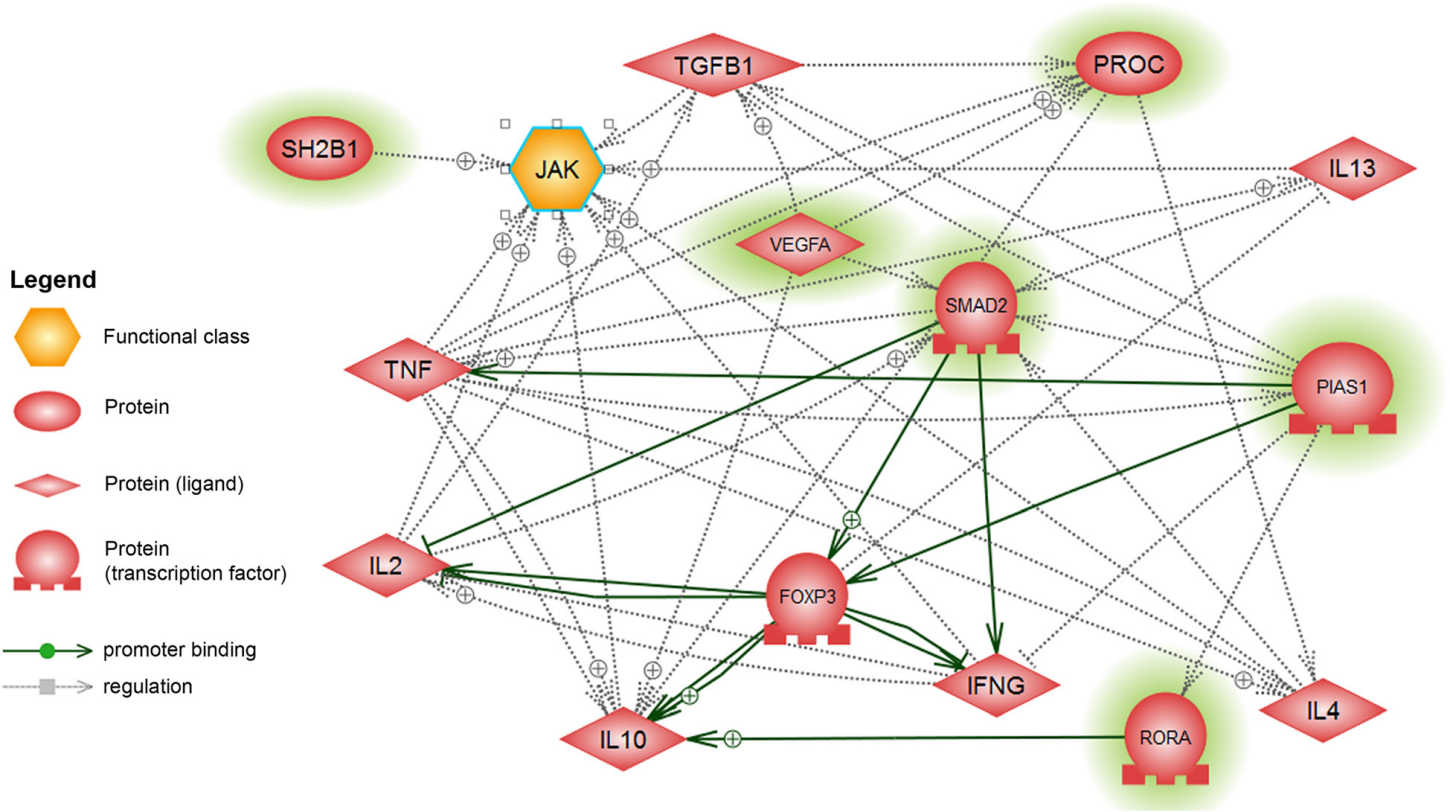
Interactions between cytokines and selected genes differentially expressed in AD and healthy dogs. Detailed network of interactions generated using Pathway Studio analysis between genes showing differences in expression in peripheral blood nuclear cells of AD and healthy dogs (green highlights) and cytokines investigated in this study

In the last stage of our study we performed a microarray analysis in order to compare the transcriptomic profile of peripheral blood nuclear cells in AD and healthy dogs. Even though we did not detect any changes in the mRNA expression level of investigated cytokines, the genes showing differential expression between AD and healthy dogs were directly or indirectly connected with regulation of T lymphocytes lineages differentiation and synthesis, as well as secretion of the aforementioned cytokines. Pathway studio analyses enabled us to find the interactions between the differentially expressed genes and cytokines investigated in the present study (Fig. 3).

Among the differentially expressed genes which showed the highest number of interactions with other genes was *VEGFA* encoding vascular endothelial growth factor (isoform A). VEGF is known to be one of the principle mediators of angiogenesis, fibroblast stimulation and tissue remodeling in allergic conditions. It may also stimulate inflammatory cell recruitment, enhance antigen sensitization and appear crucial for adaptive Th-2 inflammation [53]. A few studies demonstrated the connection between VEGF synthesis and human AD skin lesions [54]. VEGF may play a role in the pathogenesis of AD and be involved in regulation of AD lesions development acting possibly in the persisting erythema and edema by prolonged capillary dilatation and hyperpermeability [54]. Koczy-Baron et al. [55] have shown a significant increase in VEGF plasma levels in human AD patients in comparison to controls. However, these authors did not find any correlation between the plasma levels of VEGF and the number of cells that contribute to the secretion of this growth factor (mast cells, platelets). In our study the expression of *VEGFA* was decreased in peripheral blood nuclear cells of cAD patients. It is worth noting that the difference in *VEGFA* expression was observed despite similar numbers of blood elements (platelets, white blood cells) in both investigated groups of dogs (data not shown). It is possible that the role of VEGF is predominant at the site of inflammation (skin lesions) and blood levels of this growth factor are not a suitable prognostic marker of AD. Similar conclusion was stated in the work of Koczy-Baron et al. [55]. Interestingly, *VEGF* expression is stimulated by TGF-β1-induced signaling pathway, in which Smad2 and Smad3 become activated and form heterocomplexes with Smad4, enabling their translocation to the nucleus and function as transcription activators [52, 56, 57]. In our study plasma concentration of TGF-β1 did not differ significantly between AD and healthy dogs, whereas *SMAD2* expression was downregulated in cAD patients, correlating with the decreased expression of *VEGF* detected in AD dogs. These results are in agreement with the studies showing direct relationship between the *VEGF* expression and TGF-β1-induced Smad2 signaling [58] (Fig. 3).

*PIAS1* (protein inhibitor of activated STAT1) is another gene whose protein product is directly involved in regulation of transcriptional activity of Smad2 function. It has been shown that PIAS1 modulates activation of Smad2/4 complex and further promotes Smad2/4 mediated proliferation inhibition observed in some cancer cells [59]. Thus, downregulation of *SMAD2* expression observed in AD patients may be also connected with decrease levels of *PIAS1* mRNA.

RORα encoded by *RORA* gene was also among transcription factors differentially expressed in dogs with AD in comparison to controls. RORα belongs to a family of retinoid-related orphan receptors (RORs) that regulate gene transcription by binding to specific DNA response elements (ROREs) [60, 61]. RORs can function both as repressors and activators of gene transcription, interacting with corepressors, as well as coactivators of transcription. It has been demonstrated that a direct interaction of Foxp3 and RORα results in repression of RORα -mediated transcriptional activation [62] (Fig. 3). Studies with the use of stragger (sg/sg) mice, which show a congenital deficiency of the RORα (RORα^sg/sg^), demonstrated that these mice had significantly smaller spleen and the thymus, suggesting that RORα may have a role in regulation of thymopoiesis and lymphocyte development [63]. In fact, the number of mature T and B lymphocytes was significantly reduced in RORα^-/-^spleen, indicating a significant role of RORα in lymphocyte development. It has been shown that Th17 differentiation is dependent on RORs, as RORs induce the expression of *IL-17* cytokine gene [64]. On the other hand, Delerive et al. [65] demonstrated that RORα1 negatively regulated the inflammatory response by interfering with NFkB signaling pathway in primary aortic smooth muscle cells. RORα1 belongs to transcription factors which induce the expression of IkB, the major inhibitory protein of NFkB activity. Since *TNF-*α expression is induced by NFkB signaling pathway, RORα1 actions contribute to attenuation of TNF-α-induced inflammatory response [65]. In our study the plasma concentration of TNF-α significantly increased, whereas *RORA* expression was downregulated in AD dogs. Furthermore, it has been recently reported that RORα is involved in transactivation of IL-10 promoter boosting the generation of protective Tr1 cells [66]. Our results relate to these recent findings, demonstrating that decreased concentration of IL-10 in dogs with AD coincided with downregulation of *RORA* expression.

Increased levels of TNF-α in AD dogs may also be connected with downregulation of *PROC* gene, which codes for protein C, a zymogen whose active form (APC – activated protein C) plays an important role in regulating anticoagulation, inflammation and cell death [67]. Several studies have demonstrated that TNF-α inhibits activation of protein C, thereby affecting the coagulation process [68–70].

Among the investigated cytokines IL-2, IL-10 and INF-γ showed lower concentration in the blood plasma of AD dogs than in healthy animals. These cytokines act via JAK/STAT signaling pathway, in which SH2B1 (SH2 domain-containing protein) protein is recognized to play a role of a potent activator of JAK2 kinase [71]. Upon ligand binding to cytokine receptors, JAKs phosphorylate themselves and their associated receptors, thereby providing multiple binding sites for signaling proteins containing SH2 or other phosphotyrosine-binding domains [72]. SH2B1 was shown to be a potent activator of JAK2 associating with JAK2 via its SH2 domain, and thereby increasing the phosphorylation of JAK2 and its downstream targets belonging to the family of STAT transcription fatcors [71, 72]. Downregulation of *SH2B1* gene expression detected in dogs with AD can be connected with the decreased levels of cytokines, which act as ligands activating the JAK/STAT signaling pathway in the immune cells of peripheral blood.

Results of the presented microarray experiment, analyzing the transcriptomic profile of peripheral blood nuclear cells in AD and healthy dogs, are not in direct correlation with the previously published studies comparing the profiles of gene expression in biopsies of lesional skin of cAD patients and normal skin samples derived from healthy dogs [15–17]. It is evident that more pronounced differences in gene expression were recognized in the cutaneous tissue, which is the direct site of inflammatory processes occurring in AD. Authors of the aforementioned studies observed increased expression of genes involved in inflammation, wound healing, immune response [15, 17], associated with alternatively activated monocyte-derived cells, IL-1 and interferon signaling pathways [16], as well as playing a role in apoptosis, barrier formation and transcriptional regulation [15]. All genes identified in our study were downregulated in AD dogs, and the protein products of differentially expressed mRNAs are involved in transcriptional regulation (transcritipon factors: Smad2, RORα) or signal transduction (VEGF, SHB21, protein C). Juxtaposing the data obtained in the present study with results published previously suggests that transcriptomic profile of peripheral blood nuclear cells does not fully reflect the inflammatory processes which are primarily induced in the cutaneous tissue. Most probably it is caused by recruitment of the activated immune cells to the site of inflammation, thus the significant changes in gene expression will be noted especially in the lesional skin. Nevertheless, obtaining biopsies from dogs with severe AD symptoms is often problematic, and blood samples are still regarded as the valuable source of information about the state of the organism. Thus, further investigation should be done to evaluate the role of detected genes in canine atopic dermatitis.

## Conclusions

In the present study dogs with atopic dermatitis showed increased number of CD8+ T cells in peripheral blood, which may suggest that in addition to the commonly accepted role of the imbalance between Th1 and Th2 cells in the immune response during AD, Tc lymphocytes may also significantly contribute to the development of the immunoinflammatory response. Furthermore, observed increase in IL-13 concentration in the blood plasma of AD dogs in comparison to healthy animals, and an insignificant difference in the level of IL-4 between healthy and atopic individuals, support the hypothesis about the role of IL-13 as a more important mediator of the physiological changes induced by allergic inflammation. High concentrations of TNF-α detected in plasma of atopic dogs additionally confirmed the ongoing allergic inflammatory response in cAD patients. Although the number of detected Treg cells was higher in AD dogs than in healthy controls, the increased levels of TNF-α indicates the functional insufficiency of Treg cells in patients with AD, which may also explain the observed lower concentrations of IL-10 and TGF-β1 in the plasma of atopic dogs. Finally, microarray analysis of the difference in transcriptomic profile of peripheral blood nuclear cells of AD and healthy dogs revealed 59 genes downregulated in AD dogs. The list of differentially expressed genes did not include any cytokines taking part in allergic inflammation; however the function of identified genes was directly or indirectly connected with regulation of T lymphocytes lineages differentiation and synthesis, as well as secretion of the aforementioned cytokines.

Observed changes in the levels of chosen cytokines (especially IL-13) and the number of immune cells (e.g., CD8+ lymphocytes and Tregs) in peripheral blood of AD dogs encourage further investigations of possible correlations between cytokines profiles and particular stage of atopic dermatitis development. Such studies utilizing larger number of dogs may reveal potential blood markers helpful in AD diagnosis and determining adequate treatment of this disease.

## Additional file

Additional file 1: Table S1. Concentration of cytokines in plasma of dogs with atopic dermatitis and healthy dogs. (DOCX 12 kb)
