# Supplementary material for: Lymphocytic, cytokine and transcriptomic profiles in peripheral blood of dogs with atopic dermatitis
Source: BMC Vet Res. 2016 Aug 23;12(1):174. doi: 10.1186/s12917-016-0805-6 (PMC4995625; doi:10.1186/s12917-016-0805-6)
Supplement: Additional file 1: Table S1. — Concentration of cytokines in plasma of dogs with atopic dermatitis and healthy dogs. (DOCX 12 kb) [file 12917_2016_805_MOESM1_ESM.docx]

**Table S1.** Concentration of cytokines in plasma of dogs with atopic dermatitis and healthy dogs.

| Cytokines | Range of detected cytokines levels | | mean±SEM  (pg/ml) | | Number of dogs in which the cytokine was detected | | P value |
| --- | --- | --- | --- | --- | --- | --- | --- |
|  | AD | control | AD | control | AD | control |  |
| **IL-4** | 51.6 -278.8 | 48.3-196.7 | 140.3 ± 12.08 | 134.8 ± 16.33 | all | all | 0.8017 |
| **IL-13** | 56.7- 498.3 | 51.0-410.0 | 280.6 ± 29.81 | 143.5 ± 48.02 | all | all | 0.0216 |
| **IFN-γ** | 0.0-215.0 | 0.0-140.0 | 142.5 ± 75.5 | 105.0 ± 35.00 | 2 | 2 | 0.6872 |
| **IL-2** | 0-17.2 | 0.0-45.0 | 5.07 ± 2.12 | 16.48 ± 5.54 | 9 | 7 | 0.0533 |
| **TNF-α** | 58.0-166.0 | 22.0 -112.0 | 95.4 ± 6.48 | 65.0 ± 11.33 | all | all | 0.0214 |
| **IL-10** | 0.0- 417.6 | 12.0-610.0 | 62.5 ± 25.24 | 221.6 ± 85.94 | 19 | all | 0.0272 |
| **TGF-β1** | 35,1-731,7 | 141.6-615.0 | 178.4 ± 47.51 | 344.3 ± 58.96 | all | all | 0,5450 |

All concentrations are in pg/ml.
